# A model of muscle atrophy based on live microscopy of muscle remodelling in *Drosophila* metamorphosis

**DOI:** 10.1098/rsos.150517

**Published:** 2016-02-10

**Authors:** Yadav Kuleesha, Wee Choo Puah, Martin Wasser

**Affiliations:** 1 Imaging Informatics Division, Bioinformatics Institute (BII), Agency for Science, Technology and Research (A*STAR), 30 Biopolis Street, no. 07-01 Matrix, Singapore 138671, Republic of Singapore; 2 School of Computer Engineering, Nanyang Technological University, N4-2A-05, Nanyang Avenue, Singapore 639798, Republic of Singapore

**Keywords:** muscle atrophy, muscle remodelling, *Drosophila* metamorphosis, live imaging, autophagy, nuclear migration

## Abstract

Genes controlling muscle size and survival play important roles in muscle wasting diseases. In *Drosophila melanogaster* metamorphosis, larval abdominal muscles undergo two developmental fates. While a doomed population is eliminated by cell death, another persistent group is remodelled and survives into adulthood. To identify and characterize genes involved in the development of remodelled muscles, we devised a workflow consisting of *in vivo* imaging, targeted gene perturbation and quantitative image analysis. We show that inhibition of *TOR* signalling and activation of autophagy promote developmental muscle atrophy in early, while *TOR* and *yorkie* activation are required for muscle growth in late pupation. We discovered changes in the localization of myonuclei during remodelling that involve anti-polar migration leading to central clustering followed by polar migration resulting in localization along the midline. We demonstrate that the Cathepsin L orthologue *Cp1* is required for myonuclear clustering in mid, while autophagy contributes to central positioning of nuclei in late metamorphosis. In conclusion, studying muscle remodelling in metamorphosis can provide new insights into the cell biology of muscle wasting.

## 1. Introduction

The maintenance of skeletal muscle mass and strength is critical for mobility and metabolism. Apart from their contractile properties, skeletal muscles serve as reservoirs of amino acids [1]. Skeletal muscles are a tissue of high morphological and physiological plasticity. Resistance exercise can lead to increased muscle mass (hypertrophy) and strength, while nutrient starvation induces protein degradation and loss of muscle mass (atrophy). Atrophy and hypertrophy result from reversible changes in muscle fibre size, but not numbers. The failure of mechanisms that regulate atrophy may lead to irreversible muscle wasting. The two most common types of muscle wasting are sarcopenia, the age-related loss of skeletal muscle mass and function, and cachexia, a metabolic syndrome associated with diseases such as cancer, heart failure and HIV [2]. In healthy individuals, muscle mass and strength can normally be improved through exercise and a healthy diet. However, these treatments are not always feasible or effective in patients with muscle wasting conditions. The need for novel pharmacological interventions has motivated research in the molecular mechanisms of muscle wasting.

Skeletal muscle size is determined by the ratio between synthesis and degradation of sarcomeric proteins. Protein synthesis and cell growth are activated by a signalling cascade consisting of insulin-like growth factor-1, the kinase Akt1 and the mammalian target of rapamycin (mTOR). mTOR stimulates protein synthesis through phosphorylation of the ribosomal S6 kinase (S6k) and the eukaryotic initiation factor 4E-binding protein (4EBP1) [3,4]. mTOR is positively regulated by the GTPase Ras homologue enriched in brain (Rheb) and inhibited by the proteins of the tuberous sclerosis complex TSC1 and TSC2 which are triggered by a signalling pathway involving Myostatin, Smad3 and the transcription factors of the FoxO family. Protein degradation is mediated by two processes, the ubiquitin proteasome system [5] and the autophagy lysomal pathway [6]. In autophagy, proteins and organelles get encapsulated into membranous vesicles called autophagosomes that fuse with lysosomes, resulting in degradation of their cargo. The Hippo pathway which controls organ size in eukaryotes [7] has also been implicated in regulating the size of skeletal muscles in mice [8,9].

Besides disease, ageing and inactivity in mammals, developmental processes in arthropods stimulate atrophy of skeletal muscles. In the moth *Manduca sexta*, the intersegmental muscles of larvae undergo atrophy during metamorphosis prior to eclosion [10]. During moulting in decapod crustaceans such as landcrabs or lobsters, claw muscles undergo a reduction in mass to facilitate the shedding of the exoskeleton [11,12].

The fruit fly *Drosophila melanogaster* is another model to study muscle growth and atrophy in the context of animal development [13]. A genome-wide RNAi screen in *Drosophila* identified 2785 muscle-specific genes, many of which are evolutionarily conserved and implicated in human muscle diseases [14]. Skeletal muscles are formed in embryogenesis through the fusion of founder cells with fusion-competent myoblasts [15]. During 5 days of larval development, muscle fibres grow up to 50-fold [16]. During metamorphosis, which transforms larvae into adult flies, larval muscles follow two main fates. In response to ecdysone, most muscles undergo cell death. A second population of persistent muscles is resistant to hormonally induced histolysis and survives into adulthood. For instance, a group of thoracic muscles serves as a template for the formation of indirect flight muscles (IFMs) [17,18]. In the pupal abdomen, the alternative fates can be observed using *in vivo* imaging of muscles labelled with fluorescent proteins [19]. Dorsal external oblique muscles degenerate prior to head eversion (HE) at approximately 12 h after puparium formation. More basally located dorsal internal oblique muscles (DIOMs) are remodelled into temporary adult muscles that degenerate within 24 h of eclosion [20]. Remodelling of DIOMs involves atrophy in early and growth in late metamorphosis. We will refer to DIOMs as remodelled or persistent muscles depending on the context. The genes controlling the remodelling of abdominal muscles remain poorly understood. DIOM remodelling during metamorphosis coincides with the development of new adult abdominal muscles, which are formed from pools of myoblasts set aside in larvae [21].

Many muscle diseases are associated with an abnormal positioning of nuclei [22]. Centronuclear myopathies (CNM) are a group of genetically heterogeneous muscle disorders that share a common pathology consisting of muscle weakness, smaller fibres and nuclei located in the centre instead of the periphery of muscles [23]. Interactions between microtubules and the nuclear envelope proteins SUN and KASH play major roles in positioning nuclei within muscle cells [24,25]. The establishment of myonuclear positioning has been studied in development. In myogenic cell culture, nuclei of fused myoblasts move towards the centre of myotubes [26]. Similarly in *Drosophila* embryos, myoblast nuclei cluster upon fusion and subsequently split up to migrate to opposite poles of the differentiating muscle fibres [27]. Microtubule-associated motor proteins like dynein and kinesin are required for nuclear migration.

Previously, we demonstrated that high-speed confocal laser scanning microscopy (CLSM) could image abdominal muscle development throughout the entire 5-day period of *Drosophila* metamorphosis [28]. More recently, we introduced a workflow for the quantification of time-series data [29]. Here we extend this approach to identify and characterize genes and processes involved in muscle remodelling. We show that the TOR pathway and autophagy control changes of muscle fibre size in metamorphosis. Time-lapse imaging revealed that developmental muscle atrophy coincides with changes in myonuclear localization that resemble nuclear migration in early myogenesis.

## 2. Material and methods

### 2.1 *Drosophila* stocks

We used the UAS-GAL4 system [30] for targeted expression of fluorescent reporter genes, small hairpin (sh) RNAs and effector proteins in muscles. Mef2-GAL4 served as a muscle-specific driver [14]*. MHC-tau-GFP* [31] was used to label cytoplasm and *UAS-histone 2Av-mKO* [28] nuclei of muscles. All UAS-shRNA strains were derived from the Transgenic RNAi Project (TRiP) collection [32] and obtained from the Bloomington Drosophila stock centre. The UAS-shRNA and UAS-effector transgenic lines used for gene perturbation in our pilot screen are listed in the electronic supplementary material, table S1.

The *pUAS-Cp1-mKO2* reporter construct was cloned using the Gateway recombination system (Invitrogen). To visualize lysosomes, we fused the *Cp1*cDNA with a C-terminal fragment encoding monomeric Kusabira orange 2 (mKO2) [33]. The PCR amplified mKO2 ORF from the *pmKO2-S1* vector (MBL International, Woburn, MA, USA) and the Gateway cassettes were inserted into pUAST to create the *pUAST-mKO2-GWC-mKO2* destination vector. The *Cp1* ORF was amplified from the LP06554 cDNA obtained from the Drosophila Genomics Resource Center (Bloomington, IN, USA) and fused in frame with mKO2. To study Cp1-mKO2 expression in muscles, an insertion of *pUAS-Cp1-mKO2* on the third chromosome was combined with the *Mef2-GAL4* driver by meiotic recombination.

In our gene perturbation experiments, we crossed females of the reporter line *MHC-tau-GFP/FM7-GFP; Mef2-GAL4, UAS-histone-mKO/TM6B Tb* with males of the *UAS-GeneX-cDNA* or *UAS-GeneX-shRNA* lines. From the progeny, we selected non-*Tubby* prepupae expressing both fluorophores (e.g. MHC-tau-GFP/+; Mef2-GAL4, UAS-histone-mKO/UAS-GeneX-shRNA) for inspection of muscle phenotypes. For convenience, we will refer to these animals as Muscle-GO-GP (GO = Green + Orange live reporter, GP = Gene perturbation). The samples were examined using an Olympus MVX10 fluorescence macrozoom microscope (Olympus, Japan). The *UAS-Chro-shRNA* construct (TRiP#GL00503, B-36084) when crossed with Muscle-GO-GP displayed no abnormalities of muscles development, eclosion and ability to fly and was, therefore, used as control throughout this study.

### 2.2 Screening for muscle phenotypes using macrozoom microscopy

Usually, 20 Muscle-GO-GP prepupae were arranged in four groups of five samples on plastic Petri dishes with their dorsal side facing up. We recorded images at daily intervals using an Olympus MVX10 macrozoom microscope equipped with a DP73 digital CCD camera and cellSens acquisition software provided by the same manufacturer. Fields of view were recorded twice with filters for green and orange fluorescence, a zoom factor of 1.25, all of which resulted in digital colour images (TIFF or PNG format) of 2400×1800 pixels and a pixel size of 2.41μm per pixel. Phenotypes were assessed by visual inspection of individual images or montages.

### 2.3 Time-lapse confocal microscopy of metamorphosis

The protocol for sample preparation and time-lapse imaging of *Drosophila* pupae was previously described [29]. Samples were collected at the white pupal stage, rinsed with water to remove the fly food from their surface and inspected under a macrozoom fluorescence microscope to confirm expression of both reporter genes. Up to 30 prepupae were positioned on an uncoated 32 mm diameter glass bottom dish (MatTek, Ashland, MA, USA), with the dorsal side directed towards the bottom of the dish. The live samples were mounted in CyGEL (Biostatus Ltd, Leicester, UK) to restrict their movement during imaging. HE leads to compression and posterior shift of the abdomen. As our goal was to view the dorsal sides of pupal abdominal segments 1–5 during live imaging, prepupae had to be placed in such a way that the anterior border of their third abdominal segment was adjacent to one side of the field of view. We used a Zeiss LSM 5 Live (Carl Zeiss, Jena, Germany) inverted line scanning confocal microscope equipped with a motorized XY scanning stage to perform multi-location time-lapse imaging. Three-dimensional time-lapse image acquisition was carried out for 5 days at 30 min intervals (240 time points per sample) using a 10×/0.3 EC-Plan-Neofluar M27 air objective, at a scan zoom of 0.5. The two colour channels were recorded sequentially; channel 1 with an excitation laser of 488 nm, band path (BP) filter 500–525; channel 2 with 532 nm laser line, BP 560–675. Image stacks containing 35–40 optical slices were collected at 13.2 μm intervals. Each optical slice had a frame size of 1024×1024 pixels with a pixel size of 1.25 μm. The manufacturer's ZEN 2008 software was used for image acquisition, with the built-in multi-time-series macro controlling repetitive scanning of multiple locations. The temperature of the microscope room was set to 22°C. To acquire high-resolution stacks of selected muscles, samples were prepared as above, monitored with the macrozoom microscope and imaged at daily intervals using a 40×/1.3 EC-Plan-Neofluar oil DIC objective and a zoom factor of 0.5. The resulting stacks had voxel sizes of 0.31×0.31×0.48 μm (*x*, *y*, *z*).

### 2.4 Image analysis pipeline

We previously introduced a pipeline for the visualization and quantification of *in vivo* microscopy data [29]. Using the TLM-Converter custom software [34], we concatenated the image stacks stored in 8-bit LSM format to create one three-dimensional time-lapse ICS file per sample with sizes ranging from 17 to 19 GB. Three-dimensional stacks in ICS format were converted to maximum intensity projections (MIPs) to generate two-dimensional time-lapse images which were saved as multi-page TIFF files. Uncompressed TIFF files of 240 time points had sizes of 737 MB. TIFF files could be compressed over 20-fold using JPEG compression without noticeable degradation in image quality.

Using MIP time-lapse datasets as inputs, we quantified morphological changes of individual muscle fibres in three major steps. In step 1, we entered experimental parameters, such as genotype, spatial resolution and the intervals between time points. We defined the onset of HE as the temporal reference point for comparing different datasets. Step 2 involved the detection, feature extraction and annotation of regions of interest (ROIs) corresponding to muscles. Segmentation was carried out manually by drawing a contour around the muscle fibre. Subsequently, the medial axis (MA) was drawn inside the contour to measure muscle length. Average diameter was calculated by sampling equidistant points (here every 5 pixels) along the MA and determining the length of orthogonal lines towards the nearest contours on each side of the MA. Throughout the text, the diameter refers to the average length of orthogonal lines along the MA. Finally, we assigned unique IDs (e.g. DIOM 3L for the remodelled muscle in third and left abdominal hemi-segment) to track muscles and determine dynamic features. All information about experiments, images and ROIs were stored in a MySQL relational database. In step 3, we performed a time-series analysis of muscle phenotypes, comparing the features (area, mean diameter, length) of either individual or populations of muscles corresponding to different genotypes. As samples sizes were in the range of 10–20 and normal distributions could not reliably be determined, we compared populations using the non-parametric Mann–Whitney *U* (MWU) test. For graphical visualization, we showed the medians with the 25%- and 75%-tiles in scatterplots juxtaposed with their corresponding *p*-values in a separate scatterplot.

The workflow for morphological quantification was implemented as a custom tool in the C++ .NET framework and was named QuaMMM (Quantitative Microscopy of Muscles in Metamorphosis). We used the following libraries: FreeImage [35] handled the processing of multi-page TIFF files, libics the import of ICS files [36]. The ALGLIB library [37] was used to perform statistical calculations such as the MWU. This implementation of the non-parametric MWU test requires a minimum sample size of 5 and returns *p*-values in the range from 1 to 0.0001. The MySQL Connector/Net handled the communication between custom software and the MySQL database server. The results obtained with QuaMMM agree with the outputs of our earlier Java/ImageJ-based tool FMAj [29]. In addition, we verified the statistical outputs of our custom tool using Excel (Microsoft) and Minitab 16 (Minitab Inc.).

High-resolution three-dimensional stacks were processed for visualization using FIJI [38]. We designed a macro to perform batch conversion from LSM to TIFF format and colourization of the two fluorescent channels. The ‘Dynamic Resclice’ tool was applied to obtain orthogonal optical sections. To create the figures in our manuscript, we used Photoshop CS3 (Adobe) and ACDSee Pro 5 (ACD Systems International Inc.).

## 3. Results

### 3.1 Workflow for the study of muscle development in metamorphosis

**Figure 1.**
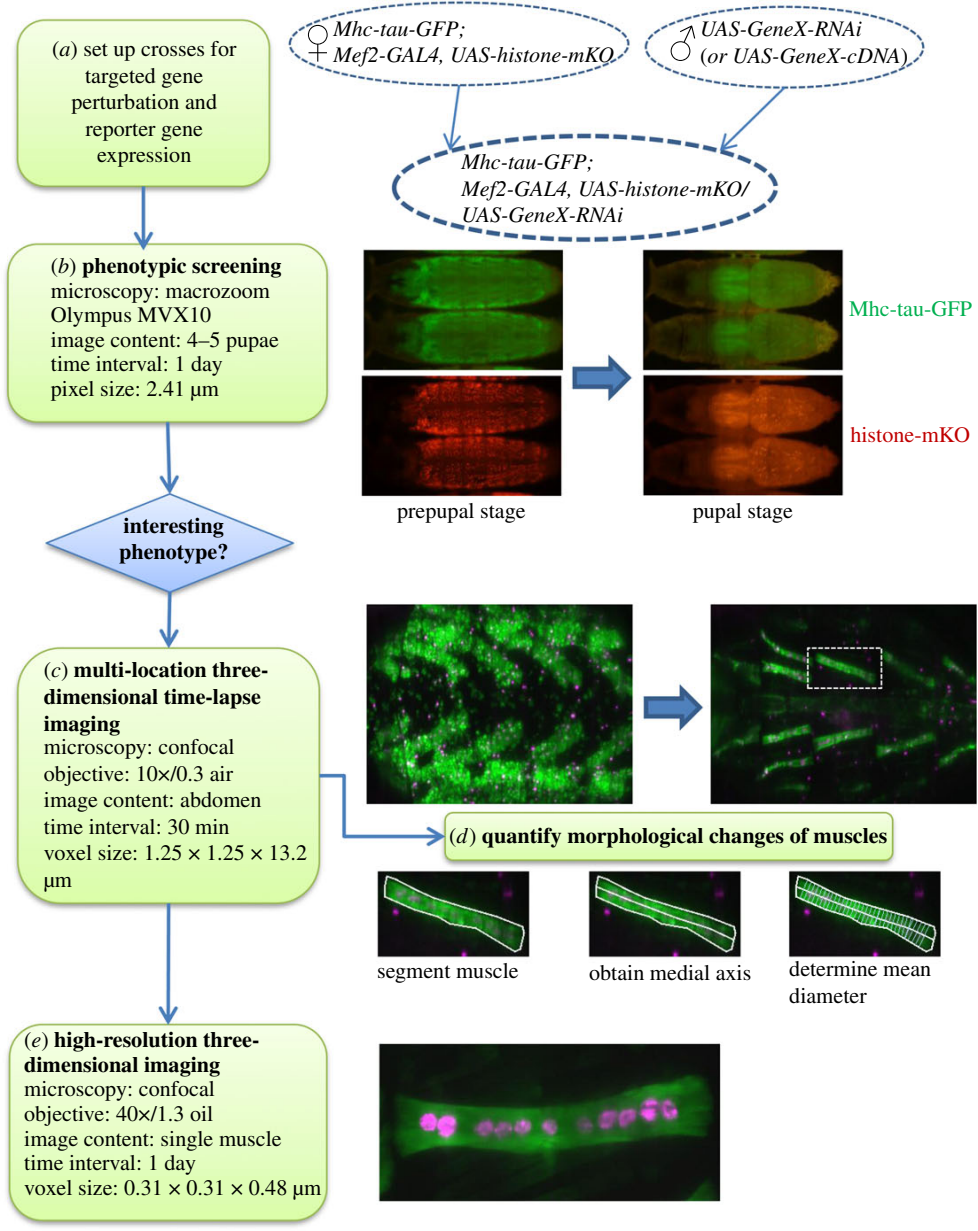
Workflow for the identification and characterization of genes involved in muscle development during *Drosophila* metamorphosis. (*a*) Flies were crossed to obtain pupae that express one shRNA and two fluorescent reporter proteins in muscles. (*b*) Live pupae were screened for abnormalities of muscle development using a macrozoom stereomicroscope at one-day intervals. (*c*) Genotypes showing interesting phenotypes were selected for three-dimensional multi-location time-lapse imaging at 30 min intervals for 5 days. (*d*) Individual muscles in maximum intensity projections of image stacks were subjected to quantitative image analysis, which consisted of manual segmentation and drawing a MA to calculate length and diameter. (*e*) To inspect individual muscles at higher spatial resolution, we acquired three-dimensional stacks using a 40×/1.3 oil lens.

To identify and characterize genes that control muscle remodelling during metamorphosis, we developed a workflow consisting of *in vivo* imaging, targeted gene perturbation and quantitative image analysis (figure 1). Females of a master stock containing three transgenes, the muscle-specific driver Mef2-GAL4, UAS-histone-mKO to label nuclei and MHC-tau-GFP to visualize the cytoplasm of muscles, were crossed to males harbouring UAS-effector constructs to drive the expression of transgenic proteins or of shRNAs for RNA interference (RNAi). The phenotypic effects of muscle-specific genes were assessed by *in vivo* microscopy using three different ways. First, to screen for interesting phenotypes, we monitored a minimum of 20 specimens per genotype at daily intervals using a fluorescence macrozoom microscope. Second, gene perturbations resulting in developmental abnormalities were subjected to multi-location, three-dimensional time-lapse CLSM from the prepupal to pharate adult stage for 5 days at 30 min intervals using a 10× air lens (electronic supplementary material, video S1). After creating time-series MIPs of three-dimensional image stacks, we segmented muscle fibres to quantify morphological changes during metamorphosis. Third, to examine selected muscles at higher spatial resolutions, we performed CLSM using a 40× oil lens.

**Figure 2.**
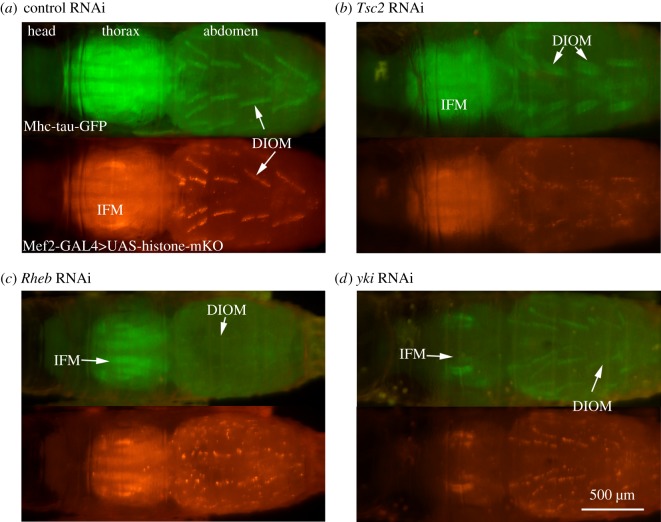
Examples of muscle phenotypes detected by macrozoom microscopy. We screened pupae expressing tau-GFP (green) and histone-mKO (red) in muscles for phenotypic effects resulting from muscle-specific gene perturbations. (*a*) Pupa expressing control-shRNA. DIOM, dorsal internal oblique muscle; IFM, indirect flight muscle. (*b*) *Tsc2* RNAi resulted in enlarged DIOMs. (*c*) *Rheb-*RNAi induced DIOMs to shrink. (*d*) *yki-*RNAi resulted in thinner DIOMs and degeneration of IFMs.

To validate our approach we performed a pilot screen with 120 publicly available UAS fly stocks, comprising 98 unique genes, targeted by 101 RNAi and 19 protein overexpression constructs. All UAS-RNAi constructs were derived from the Harvard TRiP (Transgenic RNAi Project) collection whose 21 bp target sequences are chosen to minimize off-target effects [32]. We selected genes that are involved in cell and organ growth, such as the well-studied Akt/TOR and Hippo pathways, as well as genes playing roles in autophagy, proteolysis, apoptosis and muscle differentiation. Using the macrozoom assay, we identified 19 perturbations that resulted in muscle phenotypes that were discernibly different from controls, yet did not affect development to the pharate adult stage. The abnormal phenotypes were divided into four main classes: smaller (thinner), enlarged (thicker), irregularly shaped and missing muscles (figure 2). Eighty-two genetic perturbations showed wild-type or inconclusive phenotypes; fifteen gene perturbations resulted in premature lethality in prepupal or earlier stages.

### 3.2 TOR signalling controls developmental atrophy and hypertrophy of remodelled muscles

The TOR kinase pathway positively regulates cell growth of muscles and other tissues. Qualitative inspection of images derived from the macrozoom assay suggested that inhibition of *TOR* and its activator *Rheb* led to smaller muscle fibres (figure 2*c*). To quantify changes in muscle morphology, we had previously introduced a pipeline for the analysis of CLSM images [29]. In short, the boundaries of muscles were manually segmented in MIPs of confocal images to derive shape features such as area, diameter and length (figure 1*d*). Assigning unique identities to the ROIs permitted us to track cells and monitor cellular dynamics. To compare different samples, the onset of HE was defined as temporal reference point of zero hours. Accordingly, time points are given in hours (h) after head eversion (aHE). Finally, experimental parameters, image metadata and ROI parameters (contours, feature values and annotations) were stored in a MySQL relational database for easy retrieval during statistical analysis. In the following analysis, we compared the DIOMs of the third abdominal segment at the following 13 time points: 5, 10, 15, 20, 25, 30, 40, 50, 60, 70, 80, 90 and 100 h aHE. Tables 1 and 2 list the genotypes along with the number of samples and muscles per genotype used for the quantitative analysis. About half of the samples (37 of 75) subjected to *in vivo* imaging by CLSM eclosed during the period of observation between 104 h and 110 h aHE (107:04±01:55), or approximately 4.5 days. Throughout this study, we used pupae expressing the *Chromator* (*Chro*) shRNA as controls since muscle development, eclosion rates and the ability to fly were indistinguishable from pupae that did not express any shRNA. To compare different genotypes, we determined the median feature values for each genotype time point combination and calculated significance values using the non-parametric MWU test.

**Table 1.** Time-lapse datasets used for the quantitative image analysis. The genes targeted by RNAi to study morphological changes of DIOM in abdominal segment 3. ‘*n* pupae’ refers to the number of datasets/samples per genotype, stock no. refers to the Bloomington stock centre id. Imaging experiments were carried out at a room temperature of 22°C.

gene	TRiP no.	stock no.	*n* pupae	*n* eclosed pupae	eclosion time (h.min) mean ± s.d.	eclosion rate, % (macrozoom)
*Chro* (control)	GL00503	B-36084	8	4	108.22 ± 03.42	97
*Rheb*	HMS00923	B-33966	9	7	107.34 ± 00.47	81
*TOR*	GL00156	B-35578	8	2	106.45 ± 02.28	80
*Tsc1*	GL00012	B-35144	4	2	108.00 ± 02.07	80
*Tsc2* (1)	HMS01217	B-34737	3	0	n.a.	60
*Tsc2* (2)	GL00321	B-35401	10	10	106.51 ± 00.37	100
*Atg5*	HMS01244	B-34899	6	4	107.07 ± 00.51	100
*Atg9*	HMS01246	B-34901	7	3	106.20 ± 01.15	100
*Atg12*	HMS01153	B-34675	6	1	104.00 ± 00.00	95
*Atg18*	HMS01193	B-34714	6	3	105.00 ± 00.30	100
*yorkie*(*yki*)	HMS00041	B-34067	6	1	110.30 ± 00.00	90
total			73	37	107.04 ± 01.55

**Table 2.** Time-lapse datasets used for the quantitative image analysis. The number of segmented ROIs per gene and time point.

		*n* ROIs (DIOMs) of 3rd abdominal segment scored												
gene	TRiP no.	5 h	10 h	15 h	20 h	25 h	30 h	40 h	50 h	60 h	70 h	80 h	90 h	100 h
*Chro* (control)	GL00503	15	16	16	17	15	16	17	16	16	16	16	14	14
*Rheb*	HMS00923	17	17	15	14	14	16	15	13	18	13	13	10	13
*TOR*	GL00156	16	16	16	16	15	15	15	15	16	15	15	16	13
*Tsc1*	GL00012	7	7	7	7	7	7	7	7	7	7	7	7	6
*Tsc2* (1)	HSM01217	6	6	6	6	6	6	6	6	6	6	6	6	5
*Tsc2* (2)	GL00321	18	18	18	18	18	18	16	16	17	17	13	14	16
*Atg5*	HMS01244	12	12	12	12	10	12	12	12	12	12	12	12	12
*Atg9*	HMS01246	14	15	15	14	11	14	14	14	10	14	14	14	11
*Atg12*	HMS01153	12	11	12	12	12	12	12	12	12	12	12	12	12
*Atg18*	HMS01193	12	12	12	12	11	11	10	10	12	10	12	10	12
*yorkie* (*yki*)	HMS00041	12	12	12	12	12	11	9	10	12	12	12	12	12

**Figure 3.**
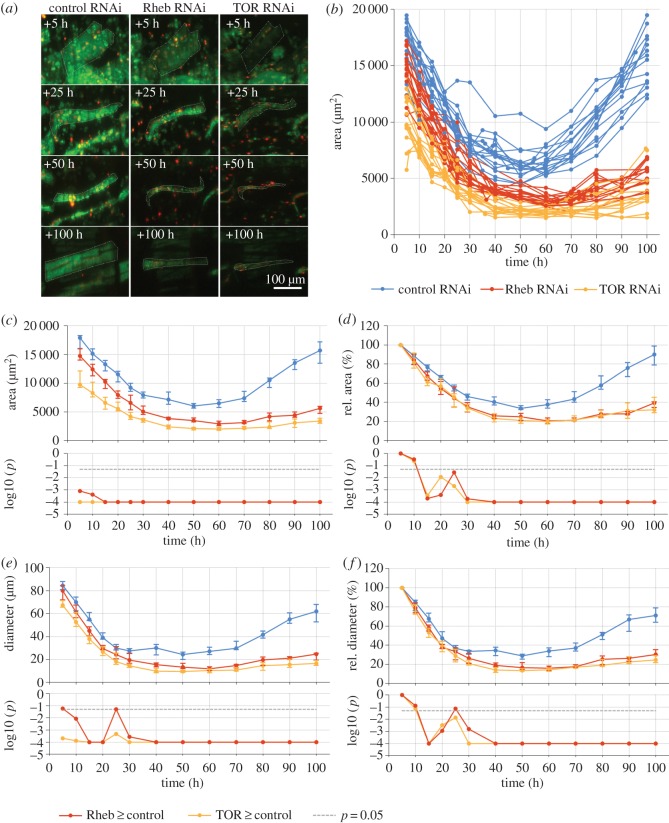
Inhibition of TOR signalling enhances atrophy in early and inhibits hypertrophy of DIOMs in late metamorphosis. (*a*) Silencing of *Rheb* and *TOR* resulted in thinner muscles. Panels compare DIOMs in the third abdominal segment at four time points aHE. (*b*) Reduction of *TOR* and *Rheb* enhanced atrophy and inhibited hypertrophy. Line plots show changes in the area of individual muscles between +5 h and +100 h. (*c*) The line chart shows the medians of the area values in (*b*) and confirms that *TOR* and *Rheb* silencing led to a significant decrease in muscle area throughout pupation. Error bars indicate the 25th and 75th percentiles. The bottom chart shows the significance values *p* (log10) of the left-tailed Mann–Whitney *U* (MWU) tests. The dashed line indicates the threshold *p*-value of 0.05. (*d*) To distinguish between phenotypic effects prior to and after head eversion, we normalized area values relative to the measurements obtained at +5 h. The diverging medians of the normalized values indicate that reduced TOR signalling increased atrophy after head eversion. (*e*) Absolute and (*f*) normalized measurements of muscle diameter further corroborate that reduced TOR signalling enhanced atrophy in early and inhibited regrowth in late pupation.

**Figure 4.**
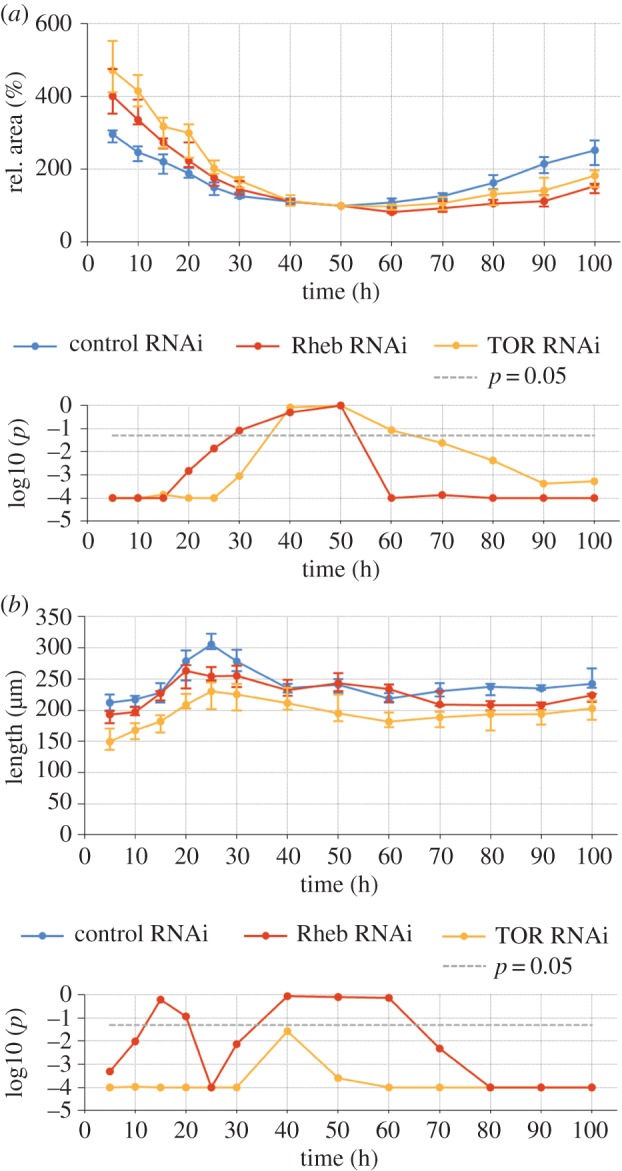
Reduced TOR signalling accelerates atrophy and inhibits hypertrophy of remodelled muscles. (*a*) The top chart shows medians of muscle area relative to time point +50 h. The steeper slope prior to and shallower slope after the reference indicate that *TOR* and *Rheb* silencing promote atrophy and inhibit growth of muscles, respectively. The bottom chart shows the significance values *p* of the two-tailed MWU tests (*Rheb*^*shRNA*^/*TOR*^*shRNA*^≠control-shRNA). (*b*) Loss of TOR signalling resulted in weaker effects on length than on diameter of DIOMs. While median diameter of *TOR*^*shRNA*^relative to control muscles declined from 80% at +5 h to 27% at +100 h (figure 3*f*), relative length slightly increased from 71% to 81%. Similarly, relative diameter of *Rheb* deficient muscles decreased from 95% to 40%, whereas relative length, being 91% at +5 h and 90% at +100 h, did not significantly change.

**Figure 5.**
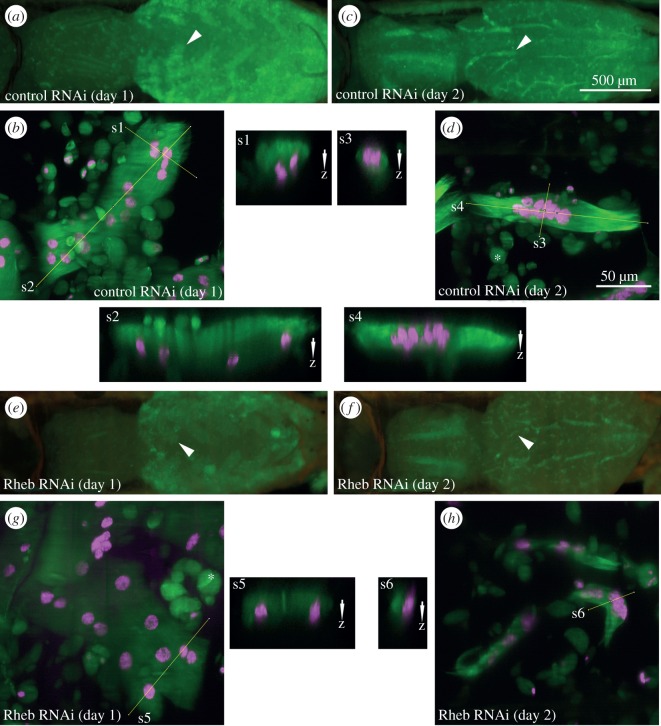
Reduction in muscle area is correlated with depletion of cell mass. Panels (*a*) and (*c*) show a control, (*e*) and (*f*) a *Rheb*-shRNA expressing pupa on the first and second day aHE imaged with a macrozoom microscope. Panels (*b*), (*d*), (*g*) and (*h*) show MIPs of three-dimensional confocal stacks of DIOMs in abdominal segment 2 (arrow heads in the corresponding pupae). Muscles were labelled with tau-GFP (green) and histone-mKO (magenta). Panels s1–s6 show orthogonal slices of the image stacks. (*b*) Muscle area decreased 2.4-fold from 8755 μm^2^ to (*d*) 3596 μm^2^, while mean depth decreased from 37.0±8.0 μm (s2) to 27.2±3.3 μm (s4), demonstrating that loss in area is correlated with depletion, not redistribution, of cell mass. (*g*) The area of the *Rheb*^*shRNA*^ DIOM decreased 5.4-fold from 14 643 μm^2^ to (*h*) 2697 μm^2^, while mean depth changed negligibly from 30.2± 3.0 μm (s5) to 30.6±3.9 μm (s6), arguing against the idea that reduced TOR signalling may induce a redistribution of cell mass. The 500 μm scale bar in (*c*) also applies to *a*, *e* and *f*. The 50 μm scale bar in (*d*) also applies to *b*, *g*, *h* and s1–s6.

In control animals, changes in muscle fibre area and diameter could be divided into two phases (figure 3). From +5 to +50 h, median area declined approximately threefold from 17 925 μm^2^ to a minimum of 6038 μm^2^ and median diameter decreased by a similar magnitude from 84.4 μm to 24.4 μm (figure 3*c*). From +50 to +100 h, area increased 2.6-fold to 15 719 μm^2^ or 90.0% of the +5 h value. A similar profile was also observed for cell diameter (figure 3*e*), but not length (figure 4), indicating that remodelled muscles progress through phases of atrophy and hypertrophy while simultaneously changing their shape, position and orientation. The correlation coefficient *r* between median area and diameter was 0.969, while *r* between area and length was −0.260. To test the alternative explanation that the change in cell area might be associated with a redistribution of cell mass from the lateral to the axial dimension rather than atrophy, we acquired three-dimensional stacks at higher planar and axial resolution (figure 5). Comparing orthogonal views of the same muscles at subsequent days (figure 5*a*–*d*) showed that loss in area was also correlated with a reduction in depth, supporting the conclusion that decreased area was indeed due to loss and not simply redistribution of muscle mass.

Silencing of *TOR* and *Rheb* led to smaller DIOM sizes throughout metamorphosis (figure 3; electronic supplementary material, video S2). Muscle area (figure 3*c*) and diameter (figure 3*e*) were significantly reduced compared with controls. The minimum relative areas with respect to controls at the equivalent time points were 22% for *TOR* RNAi at +100 h and 33% for *Rheb-*RNAi at +90 h. The minimum muscle diameters relative to control-RNAi were 27% for *TOR* at +100 h and 38% for *Rheb-*RNAi at +90 h. Since the *Mef2-GAL4* driver is active in muscles from embryogenesis onwards, cell-size changes detected during pupation may be partially due to gene perturbation in earlier development, e.g. larval stage. To help us visualize phenotypic differences without cumulative effects, we normalized the feature values with respect to the first time point of +5 h (figure 3*d*,*f*). The normalized scatter plots showed that *TOR-* and *Rheb*-RNAi, compared with controls, resulted in a significant acceleration of atrophy during pupation. While median area and thickness decreased to 34% and 29% of the +5 h values in controls, the corresponding values for *TOR*^*shRNA*^ decreased to 20% and 13%, and those for *Rheb*^*shRNA*^ to 21% and 16%. In the hypertrophic phase after +50 h, reduction of *TOR* and *Rheb* affected muscle growth. DIOM area and diameter recovered to 90% and 71% in control compared to 32% and 25% in *TOR*^*shRNA*^ and 39% and 30% in *Rheb*^*shRNA*^ muscles. Normalizing values using the time point +50 h as reference further supported this conclusion (figure 4*b*). Orthogonal views of *Rheb-*shRNA expressing muscles confirmed that the enhanced cell shrinkage seen in two-dimensional projections was due to atrophy and not redistribution of cell mass (figure 5*e*–*h*).

**Figure 6.**
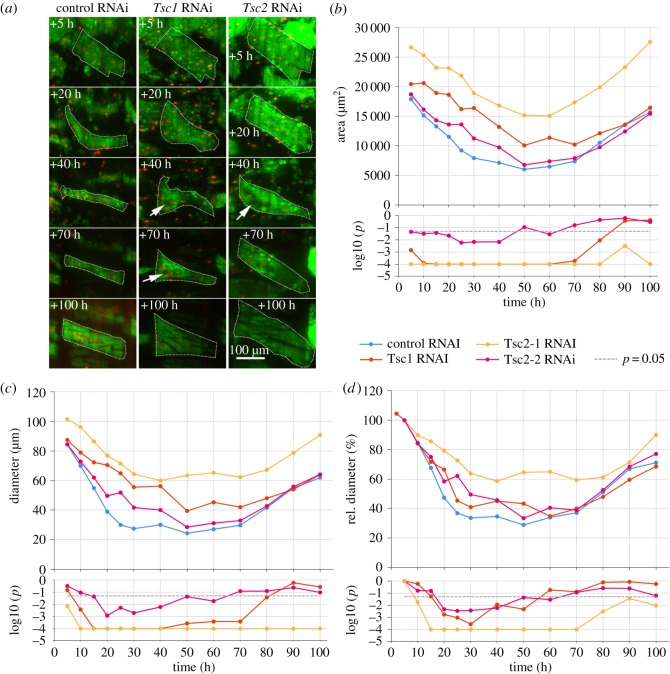
Enhanced TOR signalling inhibits atrophy of remodelled muscles. (*a*) Silencing of *Tsc1* and *Tsc2* resulted in enlarged muscles and transient appearance of vacuoles (arrows). The panels compare control, *Tsc1*^*shRNA*^ and *Tsc2*^*shRNA*^ DIOMs in the third abdominal segment at 5 time points after head eversion. (*b*,*c*) The graphs compare median area (*b*) and diameter (*c*) of *control*^*shRNA*^, *Tsc1*^*shRNA*^ and *Tsc2*^*shRNA*^ (two different alleles) muscles. The stronger *Tsc2*^*shRNA*^ allele *Tsc2-1* corresponds to the third column of panel (*a*). The bottom charts show the *p*-values (log10) of the right-tailed MWU statistical test results (*Tsc* ≤ control). All *Tsc* perturbations resulted in suppression of atrophy. The most pronounced differences in area and thickness of the weaker *Tsc1* and *Tsc2-2* alleles compared to controls were seen around +25 h to +40 h, indicating that this phase is most sensitive to silencing. (*d*) Diameter relative to +5 h was significantly decreased for the three *Tsc*-RNAi alleles from +20 h to +50 h.

 *Drosophila* *Tsc1* and *Tsc2* (*Gigas*) negatively regulate cell and organ size [39]. Consistent with this function, we observed that *Tsc1* and *Tsc2* RNAi resulted in enlarged muscles (figure 6*a*), indicating a suppression of developmental atrophy. We evaluated three *Tsc* shRNA constructs that differed in phenotypic strength with regards to increases of muscle area and diameter. The construct of TRiP ID HMS0217, referred to as *Tsc2-1*-shRNA showed the strongest phenotype, GL00321 referred to as *shTsc2-2* the weakest phenotype and the shRNA targeting the *Tsc1* gene an intermediate phenotype (figure 6*b*,*c*). Muscle area and diameter were significantly increased by *Tsc2-1*-shRNA throughout pupation, with peaks of +151% and +160% relative to controls observed at +50 h (figure 6*b*,*c*). *Tsc1* and *Tsc2-2*-RNAi caused transient muscle enlargement compared to controls. *Tsc1* RNAi led to the most pronounced atrophy repression between +25 h and +30 h when area and diameter increased approximately twofold relative to controls (+107%, +101%). The maximum transient increase of size caused by *Tsc2*-*2*-shRNA was close to 50% (+48% for area at +25 h, +48% for diameter at +30 h). *Tsc1-* and *Tsc2-2* RNAi did not cause significant cell enlargement at the beginning and end of pupation, suggesting that DIOMs were most sensitive to *Tsc* repression during the atrophic phase between +20 h and +50 h. To rule out the possibility that increased muscle size was only due to hypertrophy in the larval stages, we examined the rate of atrophy relative to early pupation at +5 h (figure 6*d*). Relative muscle diameter was significantly decreased from +20 h to +50 h for all three *Tsc*-RNAi alleles, supporting our conclusion that *Tsc* depletion slows down the rate of atrophy. *Tsc* silencing was associated with the transient appearance of vacuoles (figure 6*a*, arrows).

In conclusion, TOR signalling controls the size of DIOMs during metamorphosis. TOR activity needs to be reduced, but not switched off, to facilitate atrophy in early metamorphosis. In later metamorphosis, TOR is required to promote muscle growth. Interestingly, activators (*Rheb*) and inhibitors (*Tsc1*, *Tsc2*) of TOR appear to act simultaneously to control the magnitude of developmental atrophy.

### 3.3 Control of muscle morphology through *yorkie*

**Figure 7.**
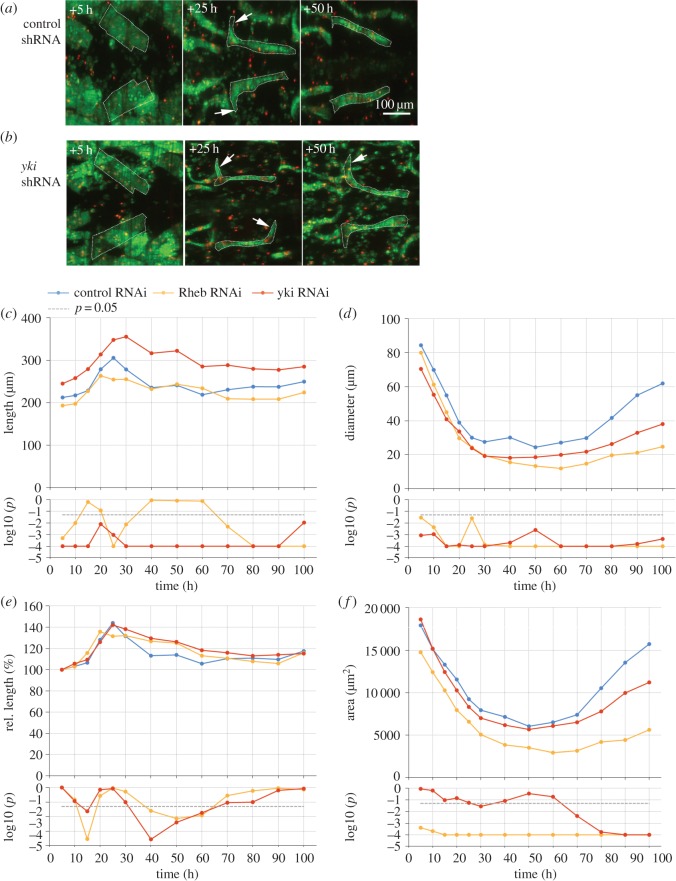
Loss of *yki* results in longer and thinner muscles. (*a*,*b*) Comparison of control and *yki*^*shRNA*^ DIOMs in the third abdominal segment at 5 time points aHE. (*c*–*f*) *yki-*, compared with control-RNAi led to significant elongation (*c*) and thinning (*d*) of DIOMs. By contrast, *Rheb* silencing decreased diameter but not length. (*a*,*b*) In the first 25 h aHE, DIOMs rotated towards the midline of the pupa, resulting in extensions that were perpendicular to the midline (arrows). (*e*) The rotations extended muscle length by approximately 40%. Subsequently control muscles shortened until +60 h as the extensions vanished. This shortening was delayed in *yki*^*shRNA*^ muscles. (*f*) *yki*-RNAi did not significantly alter muscle area until +60 h. The subsequent divergence in area compared with controls indicated that loss of *yki* inhibited muscle growth in late metamorphosis. The bottom charts show the *p*-values of the right-tailed (*c*,*e*; *yki/Rheb* ≤ control-RNAi) and left-tailed (*d*,*f*: *yki/Rheb* ≥ control-RNAi) MWU statistical test results.

Previous studies have implicated *Hippo* signalling in muscle atrophy [9]. RNAi of *yorkie* (*yki*) caused thinner DIOMs (figure 7*a*,*b*) and a degeneration of a subset of IFMs. Similar to *Rheb* and *TOR* RNAi, *yki-*RNAi caused a significant decrease of muscle diameter throughout pupation (figure 7*d*). Different from *Rheb*-RNAi, muscle length was significantly increased (figure 7*c*) and, until +60 h, area did not show differences to controls (figure 7*e*). During the hypertrophic phase after +60 h, the area of *yki* deficient DIOMs increased at a slower rate, indicating an inhibition of muscle growth. In prepupae, *yki*^*shRNA*^ DIOMs of the fourth abdominal segment were 17% longer than in control samples (table 3). The contraction of *yki*^*shRNA*^ DIOMs during HE was reduced by 8% (1.737±0.123 versus 1.893±0.135 in controls), suggesting that loss of *yki* mildly affected contractility.

**Table 3.** Loss of *yki* leads to elongation of muscle fibres. Comparison of length, diameter and contraction during HE of DIOMs in the fourth abdominal segment. For each genotype, we segmented *n*=8 muscles before (−3 h) and after (+3 h) head eversion. The *p*-values were determined using the MWU test.

feature	time point (h aHE)	control mean ± s.d.	*yki-*RNAi mean ± s.d.	*p*-value (MWU)
length (μm)	−3 h	424.2 ± 26.9	492.1 ± 24.4	0.0007
length (μm)	+3 h	224.6 ± 18.5	284.8 ± 28.2	0.0005
length ratio (%)	+3 h/−3 h	53.0 ± 3.5	57.8 ± 4.2	0.033
diameter (μm)	−3	79.9 ± 7.6	77.1 ± 8.5	0.2474
diameter (μm)	+3 h	85.9 ± 8.5	71.5 ± 13.3	0.026
diameter ratio (%)	+3 h/−3 h	108.0 ± 11.2	93.2 ± 16.0	0.0518

**Figure 8.**
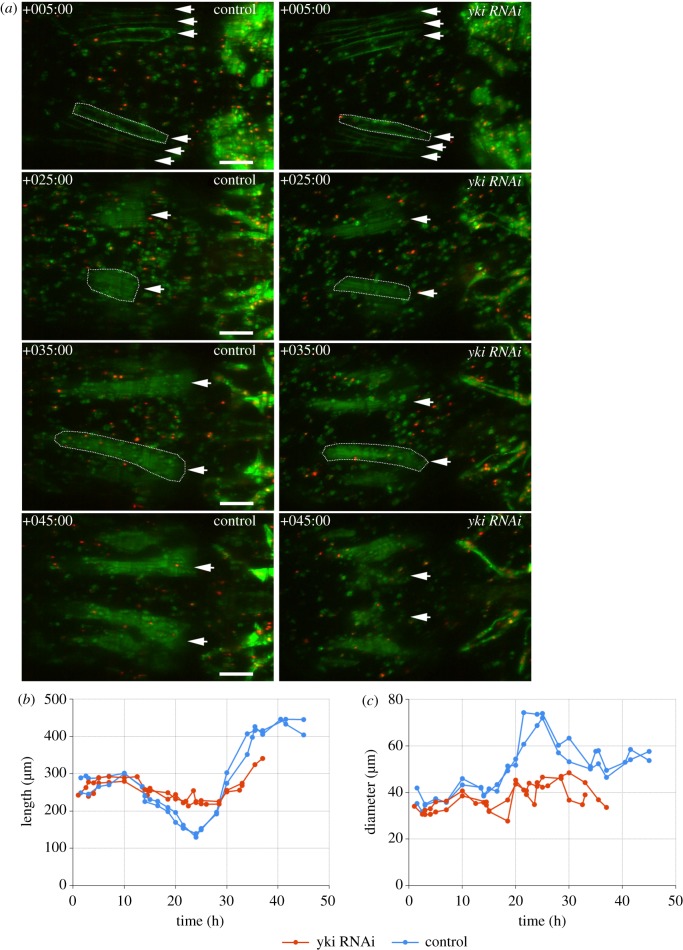
Loss of *yki* inhibits shortening of indirect flight muscle precursors and leads to their degeneration. (*a*) Dorsal views of thoracic regions of control and *yki*-shRNA expressing pupae. Arrows at +5 h aHE indicate the three larval persistent muscles that have split into six templates per hemi-segment. At +25 h, the two most dorsal templates appear as single DLM precursors (arrows) that become brighter due to fusion with myoblasts. Scale bars, 100 μm. Control DLM precursors shorten (*b*), while growing in diameter (*c*). DLMs expressing *yki*-shRNA fail to compress. At +35 h, DLM precursors elongated and at +45 h disintegrated, while the corresponding control muscle continued its growing.

Two pieces of evidence suggested that, apart from decreased contractility, a suppression of muscle shortening may contribute to muscle elongation. First, normalized length plots (figure 7*f*) showed that in the initial 25 h aHE, control and *yki*^*shRNA*^ DIOMs elongated over 40% while rotating towards the midline of the pupa (figure 7*a*,*b*; electronic supplementary material, video S3). From +25 to +60 h, control muscles shortened to 106% of their original length, whereas the rate of shortening was significantly slower in *yki*^*shRNA*^ muscles. After +60 h, the curves of both genotypes converged again. The second case of inhibited muscle shortening was observed during the development of the IFMs [40]. Three larval longitudinal oblique muscles per hemi-segment (figure 8) become templates that are infiltrated by myoblasts to differentiate into dorsal longitudinal muscles (DLMs). A previous study showed that DLMs compact in early pupation and subsequently elongate again [41]. Consistent with this report, our time-series analysis revealed that, in controls, the most dorsal DLM precursors shortened approximately twofold between +10 h and +25 h (figure 8*b*; electronic supplementary material, video S4), while increasing in thickness by a similar magnitude (figure 8*c*). From +25 h to +40 h, the differentiating DLMs elongated twofold. By contrast, the most dorsal *yki*^*shRNA*^precursors failed to compress and disintegrated during the stretching phase. The disintegration of IFM muscles was observed in all (*n*=16) *yki*-shRNA over-expressing samples recorded by macrozoom (figure 2*d*) or confocal microscopy. The DLMs derived from the more ventral templates remained intact.

### 3.4 Autophagy promotes muscle atrophy during muscle remodelling

**Figure 9.**
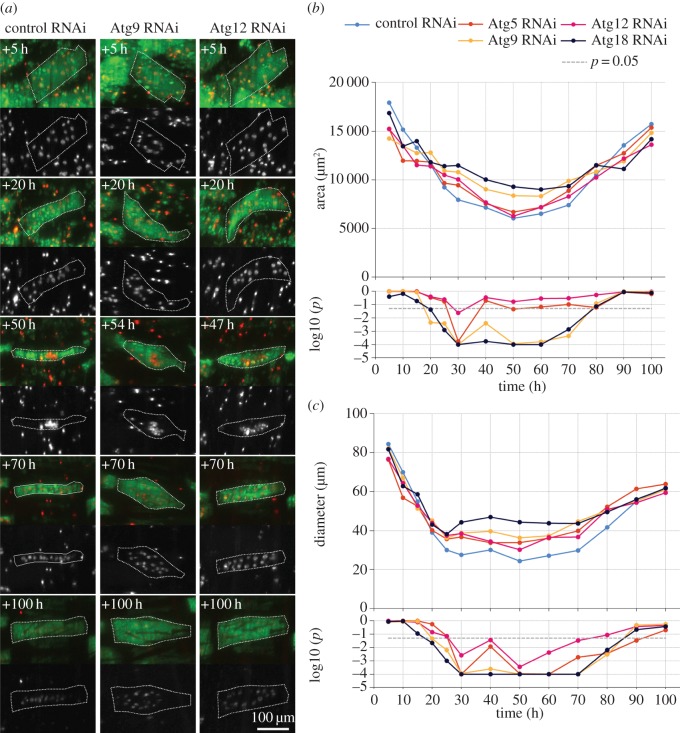
Inhibition of autophagy affects developmental atrophy and myonuclear distribution. (*a*) The panels compare *control*^*shRNA*^, *Atg9*^*shRNA*^ and *Atg12*^*shRNA*^ DIOMs in the third abdominal segment at 5 time points. Each panel shows the tau-GFP (green) labelled muscle at the top and histone-mKO labelled nuclei (white) at the bottom. Compared with controls, the thinning of *Atg*^*shRNA*^ muscle fibres is attenuated and less uniform. Note the bulging centre in the *Atg9*^*shRNA*^ muscle at +54 h aHE and later. (*b*,*c*) Differences of median muscle area and diameter indicate that reduced autophagy caused transient suppression of atrophy. RNAi of four autophagy-related genes significantly increased diameter from +30 h to +70 h, while area was significantly increased by silencing *Atg9/18* (not *Atg5/12*). The bottom charts in (*b*) and (*c*) show the *p*-values of the right-tailed (Atg ≤ control-RNAi) MWU statistical tests. (*a*) Myonuclei show three patterns of localization. In early pupation (+5 h, +20 h), nuclei were uniformly distributed. In mid pupation, nuclei migrated in an anti-polar fashion to form central clusters at around +50 h (third row). Subsequently, nuclei in control muscles moved towards the poles to adopt positions near the midline (+70 h, +100 h). By contrast, silencing of the four autophagy-related genes resulted in scattered nuclear distribution.

TOR kinase signalling promotes muscle growth by inhibiting the autophagy machinery, which sequesters proteins and organelles for lysosomal degradation. To study the role of autophagy in muscle metamorphosis, we expressed shRNAs targeting 17 different autophagy-related genes. Silencing of five genes (*Atg5, Atg9, Atg12, Atg17* and *Atg18*) resulted in thicker fibres, irregular shapes, characterized by central bulging and more scattered distributions of myonuclei (figure 9*a*; electronic supplementary material, video S5). Four genes (*Atg5, Atg9, Atg12*and*Atg18*) were studied in more detail by time-lapse CLSM. The repression of all four genes resulted in transient, significant increases of fibre diameter between +20 h and +70 h (figure 9*b*). Cell area increased significantly in response to *Atg9* and *Atg18*, but not *Atg5* and *Atg12* silencing (figure 9*c*). The largest median increases of thickness and area relative to controls were observed for *Atg18* RNAi at +50 h with values of 82% and 54%, respectively. No significant enlargements of muscle fibres were encountered at the beginning and end of the pupal stage, indicating that autophagy acts transiently to promote atrophy.

### 3.5 Myonuclear migration during muscle remodelling

**Figure 10.**
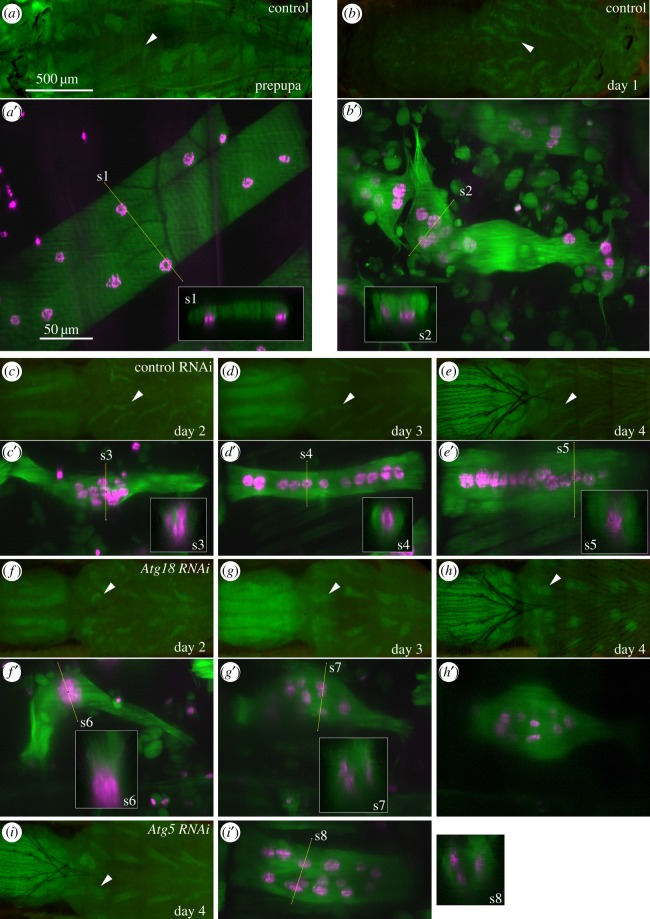
Myonuclei of DIOMs change localization during remodelling. (*a*–*i*) Dorsal views of pupae imaged using macrozoom microscopy. Arrow heads indicate DIOMs in abdominal segment 2 that were acquired by CLSM and are shown in (*a*^′^–*i*^′^) (tau-GFP, green; histone-mKO, magenta). Panels s1–s8 show orthogonal slices of the three-dimensional image stacks. (*c*–*e*) and (*f*–*h*) belong to the same control and *Atg18*^*shRNA*^ samples, respectively. (*a*^′^, s1) In prepupae, nuclei were uniformly distributed along the basal side of DIOMs. (*b*^′^,s2) On day 1 aHE, nuclei remained scattered along the basal side. (*c*^′^,s3) On day 2, nuclei clustered in the centre of muscles. (*d*^′^) On day 3, nuclei migrated towards the poles and distributed along the midline. (s4) Nuclei occupied central positions along the *z*-axis. (*e*^′^, s5) During muscle growth, nuclei remained near the midline. (*f*^′^) Despite *Atg18* deficiency and abnormal morphology, nuclei clustered in the centre. (*g*^′^,*h*^′^) On days 3 and 4, nuclei moved towards the poles and became displaced from the midline. (*i*^′^, s8) Nuclei were scattered in an *Atg5*^*shRNA*^ muscle, although size and shape were similar to the control muscle in (*e*^′^). The 500 μm and 50 μm scale bars in (*a*) and (*a*^′^) apply to all macrozoom and confocal images, respectively.

**Figure 11.**
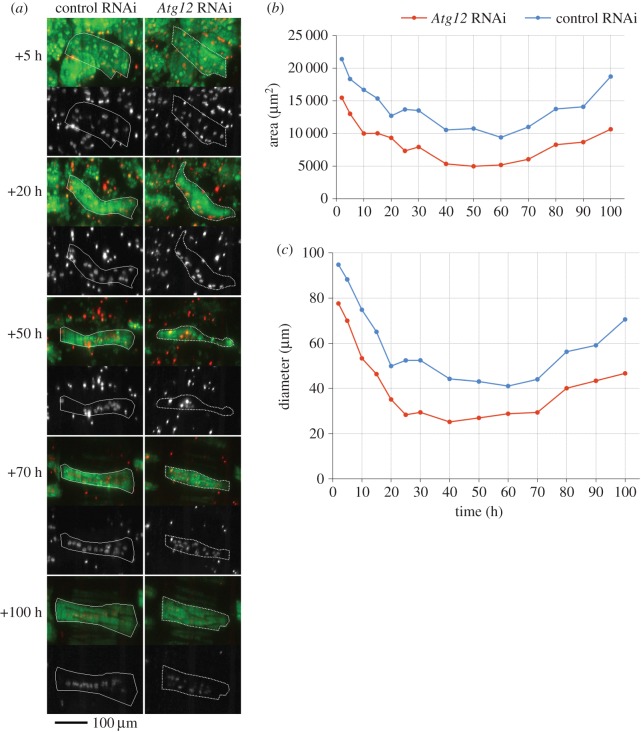
The loss of autophagy affects myonuclear distribution in late pupation. (*a*) Comparison of an above average-sized control with a below average-sized *Atg12*-shRNA expressing DIOM at 5 time points during pupation. Tau-GFP (green, top) labels the cell body, while histone-mKO (white, bottom) visualizes myonuclei. Although area (*b*) and diameter (*c*) of the *Atg12*^*shRNA*^muscle are smaller than that of the control cell throughout metamorphosis, nuclei show a scattered two-row distribution (+70 h, +100 h), while nuclei in the control muscle become arranged in a single line.

Myonuclei display reproducible changes in subcellular localization. In prepupae and early pupae, nuclei were evenly distributed along the basal side of the muscle fibre (figures 9*a* and 10*a*). This pattern remained until approximately +40 h when nuclei began to migrate in an anti-polar fashion and form one or two central clusters (figures 9*a*, third row and 10*c*). In the next 10 h, nuclei migrated away from the central clusters towards the poles to localize along the midline of muscle fibres (figures 9*a*, fourth row and 10*d*). As muscle diameters expanded in late metamorphosis, nuclei remained located along the MA, giving rise to single-row configurations. Loss of autophagy affected myonuclear positioning in later metamorphosis. RNAi of *Atg5*, *Atg9*, *Atg12* and *Atg18* caused dispersed nuclear distributions (figures 9*a* and 10*g*–*i*). Time-lapse imaging revealed that irregular morphology resulting from *Atg* silencing did not affect anti-polar migration or clustering of nuclei (figure 9*a*, third row). Instead, displacement from the midline happened during polar nuclear migration (figure 9*a*, cf. nuclear localization of control with *Atg9*- and *Atg12*-shRNA in third and fourth row). A plausible explanation of nuclear scattering is increased lateral freedom of motion, which leads to displacement from the midline. This model would predict that thicker control muscles would tend towards scattering, while thinner autophagy deficient muscles would constrain lateral movements and display single-row nuclear distributions along the midline. However, the comparison of below average-sized autophagy deficient with above average-sized control muscles contradicted this prediction (figure 11; electronic supplementary material, video S6). Despite a smaller area and diameter, nuclei in an *Atg12*^*shRNA*^ muscle were displaced from the MA, while the nuclei in a larger control muscle adopted a single line configuration. Abnormal nuclear distribution was also observed in an *Atg5*^*shRNA*^ DIOM of normal morphology and size (figure 10*i*).

**Figure 12.**
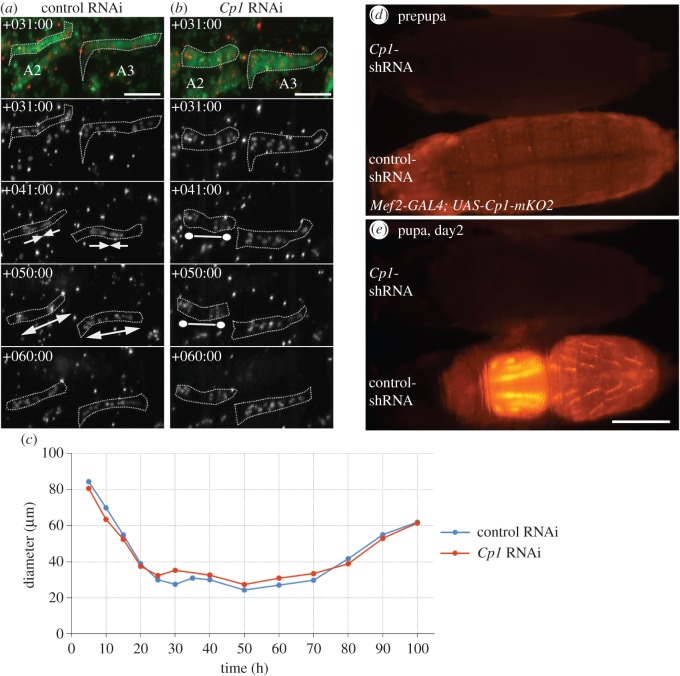
Silencing of *Cp1* inhibits myonuclear migration in mid pupation. (*a*) In control DIOMs of abdominal segments 2 and 3 (A2, A3) nuclei migrated in an anti-polar direction (+41 h aHE) followed by polar movements (+50 h). (*b*) *Cp1* RNAi repressed nuclear migration, thus preventing clustering of nuclei. The DIOM in A2 degenerated at +65 h, while the muscle in A3 persisted until eclosion. (*c*) The decreased median diameter of DIOMs in A3 indicates that *Cp*-RNAi did not inhibit atrophy. (*d*,*e*) *Cp1*^*shRNA*^ eliminated expression of a Cp1-mKO2 reporter in prepupae and pupae. Scale bars, (*a*,*b*) 100 μm and (*e*) 500 μm.

We tested the effects of loss of Cysteine Proteinase-1 (*Cp1*), the fly orthologue of lysosomal Cathepsin L, on muscle size and nuclear migration. *Cp1* RNAi resulted in premature cell death of 66.7% of DIOMs scored (48 DIOMs in eight pupae). Since cell death occurred in mid pupation at +55.3 h (±13.4), we were able to study atrophy and nuclear migration. In eight pupae analysed, we did not observe discernible nuclear anti-polar or polar migration (figure 12*a*,*b*) irrespective of whether muscles survived until eclosion or not. Time-series analysis showed that muscles shrank, indicating that lack of *Cp1* did not inhibit atrophy (figure 12*c*). Using a fluorescent reporter gene expressing Cp1-mKO2, we could confirm that the shRNA constructs targeted *Cp1* as the fluorescence was completely abolished in prepupae and pupae (figure 12*d*,*e*). In summary, atrophy in remodelled muscles is accompanied by reproducible myonuclear migration and distribution. The lysosomal proteinase Cp1 is required for myonuclear migration, while autophagy helps to position along the midline of growing muscles.

## 4. Discussion

**Figure 13.**
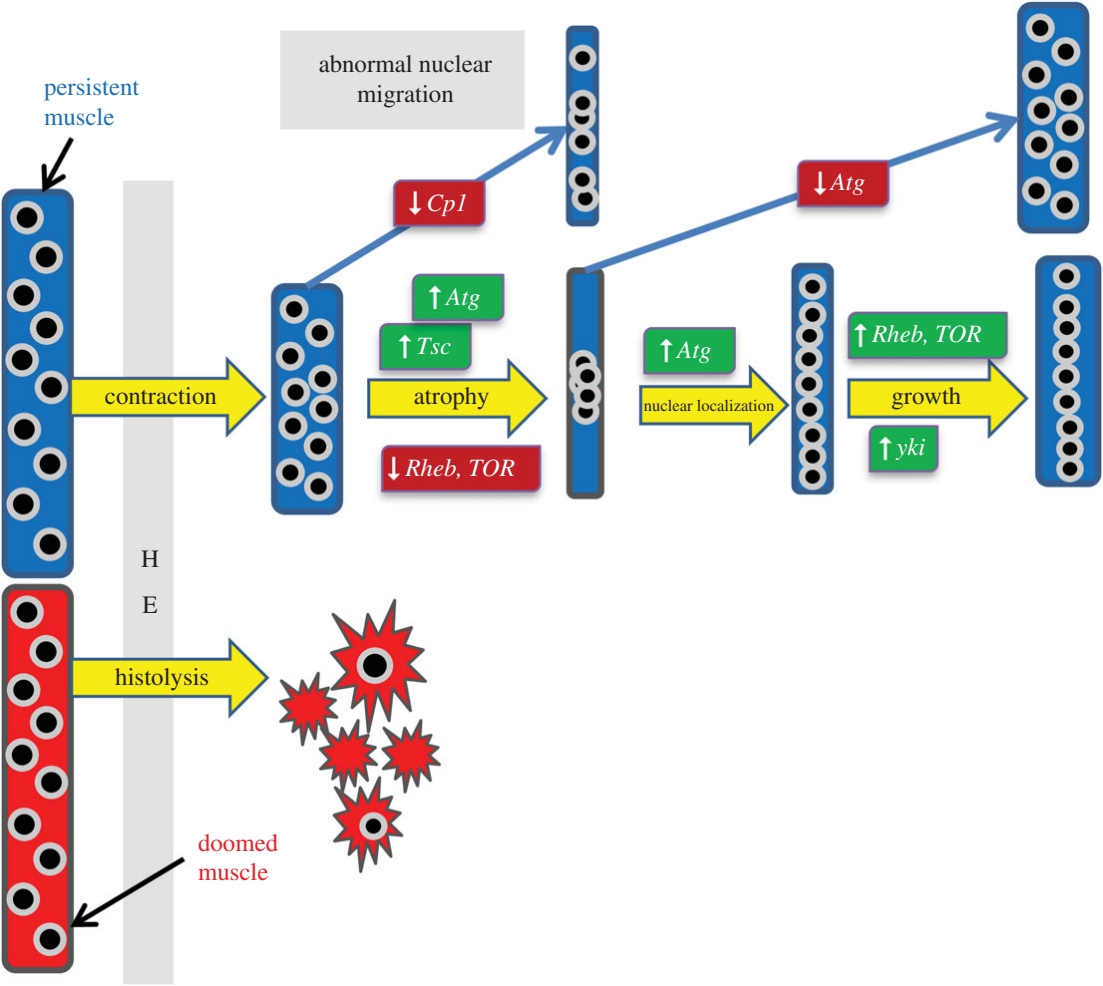
Summary of processes involved in muscle remodelling during metamorphosis. Persistent muscles contract during HE and undergo atrophy, which is activated through Tsc mediated inhibition of TOR signalling. Depletion of cell mass depends on autophagy (Atg). Muscle remodelling is accompanied by changes in myonuclear localization, which are reminiscent of nuclear migration in early myogenesis, suggesting that atrophy induces dedifferentiation into a ‘myotube-like’ state.

The goal of this study was to show the spectrum of insights quantitative microscopy can provide about the processes and genes that mediate muscle remodelling in metamorphosis (figure 13). We demonstrate that developmental muscle atrophy in flies and physiological atrophy in mammals are regulated by conserved signalling pathways. Remodelling of DIOMs can be broadly divided into two phases, an atrophic phase where area and thickness decrease threefold and a hypertrophic phase where both features recover by a factor of 2.5, each of which last around 50 h given an ambient temperature of 22°C. Our results showed that TOR signalling was involved in both phases. As expected, the loss of the TOR kinase inhibitors *Tsc1* and *Tsc2* caused an attenuation of atrophy. Not totally expected, the reduction of *TOR* and its activator *Rheb* enhanced atrophy, indicating that TOR activity needs to be reduced but not fully stopped to achieve a wild-type reduction of muscle size. *TOR* was also required in the hypertrophic phase since silencing of *TOR* and *Rheb* reduced growth rate and final muscle size. Similarities between *Drosophila* and mammals go beyond muscle size. The loss of the *Tsc* genes was associated with the transient occurrence of vacuoles, which were also reported to be caused by TSC knockouts in mouse muscles [42]. Vacuoles in muscles are associated with the accumulation of glycogen in lysosomes, as in the case of Pompe's disease [43]. Autophagy targets proteins and organelles for lysosomal degradation. Silencing of four autophagy-related genes (*Atg5, Atg9, Atg12* and *Atg18*), similar to *Tsc* knockdowns, caused the transient suppression of developmental atrophy in DIOMs, indicating that autophagy plays a major role in depletion of muscle mass. Consistent with our data, a previous study reported that autophagy played a role in shrinking larval *Drosophila* midgut cells prior to cell death [44]. A recent study implicated Yap, the downstream effector of Hippo signalling, as a positive regulator of skeletal muscle mass in mice [9]. In late metamorphosis, loss of the YAP homologue *yki*, similar to reduction of TOR signalling, resulted in the inhibition of muscle growth. The common phenotype is consistent with the finding that YAP can activate mTOR in mammalian cells [45]. In addition, our data provide evidence that *yki* may regulate muscle shape since its loss resulted in elongation by suppressing the shortening of DIOMs and DLMs. The cell death in a subset of DLMs appears consistent with the function of *yki* as a suppressor of cell death [46,47]. However, the selectivity of this phenotype remains to be determined.

We discovered myonuclear migration during muscle remodelling, suggesting a link between atrophy and nuclear localization. In prepupae and early pupae, nuclei were attached to the basal side. Towards the end of the atrophic phase (+40 h aHE), nuclei migrated away from the poles to cluster in the centre of the fibre. Subsequently, nuclei migrated in the opposite direction towards the poles to position themselves along midline. Myonuclear migration during pupation bears striking resemblance to events in early myogensis. In cultured mouse tissues, nuclei of fused myoblasts migrate towards the centre of myotubes [26,48]. Similarly in *Drosophila* embryos, nuclei of fused myoblasts first cluster and then split up to migrate to opposite poles of differentiating muscle fibres [48]. The myonuclear movements in DIOMs during metamorphosis could be interpreted as muscle fibre dedifferentiation into a myotube-like state followed by the recapitulation of an embryonic differentiation programme. The absence of nuclear migration in response to loss of *Cp1* suggests a link between lysosomal proteolysis and nuclear positioning. A plausible explanation is that *Cp1* might disrupt the attachment of myonuclei to the cytoskeleton or membrane. Furthermore, our data suggest a connection between autophagy and nuclear positioning since nuclei failed to remain near the midline during polar migration. Interestingly, knockout mice for MTM1, a gene involved in CNM, showed an impairment of autophagy in conjunction with centrally located nuclei [49,50].

Given that muscle remodelling is controlled by conserved signalling pathways, larger and unbiased genome-wide screens have the potential to identify additional regulators of cell size, shape and nuclear migration, some which may be conserved with humans. It may seem premature to claim that developmental muscle atrophy in insects is a model for muscle wasting in human ageing and disease. Nevertheless, we witnessed a couple of remarkable similarities in terms of pathology and genetic networks. Therefore, visualizing proteins and organelles in live *Drosophila* muscles during atrophy, hypertrophy and degeneration can address basic biological questions that complement research in mammalian systems.

## Supplementary Material

Supplementary Table 1.

## Data accessibility

The QuaMMM software and sample datasets are available for download at the following link: https://figshare.com/s/dad5a7c8f77b9bb4663b (doi:10.6084/m9.figshare.2059965). *Drosophila* stocks newly generated for this study have been deposited at the Vienna Drosophila Resource Center (VDRC): http://stockcenter.vdrc.at.
