# Supplementary material for: A model of muscle atrophy based on live microscopy of muscle remodelling in Drosophila metamorphosis
Source: R Soc Open Sci. 2016 Feb 10;3(2):150517. doi: 10.1098/rsos.150517 (PMC4785973; doi:10.1098/rsos.150517)
Supplement: Supplementary Table 1. [file rsos150517supp1.pdf]

Supplementary Material

Supplementary Table 1. List of gene perturbations used in this study. The UAS constructs were expressed in muscles using the Mef2-GAL4 driver along with the fluorescent reporters MHC-tauGFP and UAS-histone-mKO. Gene perturbation types included RNA interference (RNAi), protein overexpression (OE) and overexpression of dominant negative (DN) proteins. Phenotypes were evaluated using a macrozoom microscope. The column Lethality Stage refers to the proportion of prepupae/pupae undergoing developmental arrest before pupariation (BP), as prepupae (PP), as early pupae (EP) and late pupae (LP). Flightless rate refers to the proportion of eclosed flies that were unable to fly inside a large fly bottle. Rows highlighted in blue contain gene perturbations that were analysed in this study by time-lapse microscopy using a CLSM. (n.d. = not determined).

Supplementary Table 1

Gene pertubed	Biological function	Gene perturbation Type	Construct/ TRip #	Stock Number	Lethality Stage	Eclosion [%]	Flightless rate [%]	DIOM phenotype
**7B2**	Proteolysis, peptidase activator	RNAi	UAS-7B2-lhRNA (Long Hairpin JF02917)	B-27989	PP 5%	95%	0%	wildtype
**Adenosine deaminase acting on RNA (Adar)**	RNA editing	RNAi	UAS-Adar-lhRNA (Long Hairpin JF02942)	B-28311		100%	0%	wildtype
**Akt1**	Cell growth, organ size	Protein OE	UAS-Akt1	B-8191		100%	0%	wildtype
**Akt1**	Cell growth, organ size	RNAi	UAS-Akt1-lhRNA (Long Hairpin HM04007)	B-31701	LP 35%	65%	partial	inconclusive
**Akt1**	Cell growth, organ size	RNAi	UAS-Akt1-shRNA (Short Hairpin HMS00007)	B-33615	BP 100%	0%	n.d.	n.d.
**ALG-2 interacting protein X (ALiX)**	Ubiqitin dependent proteolysis	RNAi	UAS-ALiX-shRNA (Short Hairpin HMS00298)	B-33417				wildtype
**Activator of SUMO 1 (Aos1)**	Protein sumoylation	RNAi	UAS-Aos1-shRNA (Short Hairpin GL00493)	B-36074	LP 25%	75%	0%	wildtype
**Autophagy-related-1 (Atg1)**	Autophagy	RNAi	UAS-Atg1-shRNA (GL00047)	B-35177				wildtype
**Autophagy-related-10 (Atg10)**	Autophagy	RNAi	UAS-Atg10-shRNA (Short Hairpin HMS02026)	B-40859				wildtype
**Autophagy-related-101 (Atg101)**	Autophagy	RNAi	UAS-Atg101-shRNA (Short Hairpin HMS01349)	B-34360				wildtype
**Autophagy-related-12 (Atg12)**	Autophagy	RNAi	UAS-Atg12-RNAi (Short Hairpin HMS01153)	B-34675	EP 5%	95%	0%	hypertrophy
**Autophagy-related-13 (Atg13)**	Autophagy	RNAi	UAS-Atg13-shRNA *Short Hairpin HMS02028)	B-40861				wildtype
**Autophagy-related-14 (Atg14)**	Autophagy	RNAi	UAS-Atg14-shRNA (Short Hairpin HMS02025)	B-40858	PP 5%, LP 15%	80%	50%	wildtype
**Autophagy-related-16 (Atg16)**	Autophagy	RNAi	UAS-Atg16-shRNA (Short Hairpin HMS01347)	B-34358				wildtype
**Autophagy-related-17 (Atg17)**	Autophagy	RNAi	UAS-Atg17-shRNA (Short Hairpin HMS01611)	B-36918				abnormal morphology

Gene pertubed	Biological function	Gene perturbation Type	Construct/ TRip #	Stock Number	Lethality Stage	Eclosion [%]	Flightless rate [%]	DIOM phenotype
**Autophagy-related-18 (Atg18)**	Autophagy	RNAi	UAS-Atg18-shRNA (Short Hairpin HMS01193)	B-34714		100%	0%	hypertrophy
**Autophagy-related-2 (Atg2)**	Autophagy	RNAi	UAS-Atg2-shRNA (Short Hairpin HMS01198)	B-34719	BP 100%	0%	n.d.	n.d.
**Autophagy-related-4a (Atg4a)**	Autophagy	RNAi	UAS-Atg4-shRNA (Short Hairpin HMS01482)	B-35740	LP 2%	98%	78%	wildtype
**Autophagy-related-5 (Atg5)**	Autophagy	RNAi	UAS-Atg5-shRNA (Short Hairpin HMS01244)	B-34899		100%	0%	hypertrophy
**Autophagy-related-6 (Atg6)**	Autophagy	RNAi	UAS-Atg6-shRNA (Short Hairpin HMS01483)	B-35741	LP 2%	98%	0%	wildtype
**Autophagy-related-7 (Atg7)**	Autophagy	RNAi	UAS-Atg7-shRNA (Short Hairpin HMS01358)	B-34369		100%	0%	wildtype
**Autophagy-related-8a (Atg8a)**	Autophagy	RNAi	UAS-Atg8a-shRNA (Short Hairpin HMS01328)	B-34340		100%	0%	wildtype
**Autophagy-related-8b (Atg8b)**	Autophagy	RNAi	UAS-Atg8b-shRNA (Short Hairpin HMS01245)	B-34900		100%	0%	wildtype
**Autophagy-related-9 (Atg9)**	Autophagy	RNAi	UAS-Atg9-shRNA (Short Hairpin HMS01246)	B-34901		100%	0%	hypertrophy
**beta subunit of type I geranylgeranyl transferase (betaggt-I)**	Protein prenylation, RAS signalling	RNAi	UAS-betaggt-I-shRNA (Short Hairpin HMS01165)	B-34687		100%	0%	wildtype
**Bruce**	apoptosis	RNAi	UAS-dBruce-RNAi	VDRC 107620		100%	0%	wildtype
**Bruce**	apoptosis	RNAi	UAS-dBruce-RNAi	VDRC 48309		95%	0%	wildtype
**bent (bt)**	sarcomere organization	RNAi	UAS-bt-lhRNA (Long Hairpin JF01108)	B-31546	larval	0%	n.d.	n.d.
**Cadherin 99C (Cad99C)**	Cell adhesion	RNAi	UAS-Cad99C-lhRNA (Long Hairpin JF02660)	B-27510	LP 5%	95%	0%	wildtype
**Cadherin 99C (Cad99C)**	Cell adhesion	RNAi	UAS-Cad99c-shRNA (Short Hairpin HMS01451)	B-35037	LP 10%	90%	0%	wildtype
**Catalase (Cat)**	Heart morphogenesis	RNAi	UAS-Cat-shRNA (Short Hairpin HMS00990)	B-34020		90%	0%	wildtype

Gene pertubed	Biological function	Gene perturbation Type	Construct/ TRip #	Stock Number	Lethality Stage	Eclosion [%]	Flightless rate [%]	DIOM phenotype
**cathD**	autophagic cell death, proteolysis	RNAi	UAS-cathD-lhRNA (Long Hairpin HM05189)	B-28978		100%	0%	wildtype
**CG13579**	G-protein couples receptor	RNAi	UAS-CG13579-lhRNA (Long Hairpin JF03059)	B-28644		100%	0%	wildtype
**chico**	Cell size, proliferation	RNAi	UAS-chico-shRNA (Short Hairpin HMS01553)	B-36665		95%	0%	wildtype
**chico**	Cell size, proliferation	RNAi	UAS-chico-shRNA (Short Hairpin GL00525)	B-36788		95%	0%	wildtype
**Chro (Chromator)**	Interacts with EAST	RNAi	UAS-Chro-shRNA (Short Hairpin GL00503)	B-36084	LP 2%	97%	0%	wildtype
**coracle (cora)**	actin cytoskeleton, heart development	RNAi	UAS-cora-shRNA (Short Hairpin HMS01413)	B-35003		100%	no	wildtype
**Cysteine Proteinase 1 (Cp1)**	autophagic cell death	RNAi	UAS-Cp1-shRNA (Short Hairpin HMS02336)	B-41939		100%	0%	degeneration
**Cysteine Proteinase 1 (Cp1)**	autophagic cell death	RNAi	UAS-Cp1-shRNA (Short Hairpin HMS00725)	B-32932	LP 5%	95%	0%	degeneration
**dawdle**	Myostatin homolog	RNAi	UAS-dawdle-shRNA (Short Hairpin HMS01110)	B-34974				wildtype
**death executioner Bcl-2 homologue (debcl)**	apoptosis	RNAi	UAS-debcl-lhRNA (Long Hairpin JF02429)	B-27083		100%	0%	wildtype
**Drosophila inhibitor of apoptosis 1 (Diap1)**	negative regulator of apoptosis	Protein OE	UAS-Diap1	B-6657		100%	0%	wildtype
**Drosophila inhibitor of apoptosis 1 (Diap1)**	Apoptosis	RNAi	UAS-Diap1-shRNA (Short Hairpin HMS00752)	B-33957				wildtype
**Death-associated inhibitor of apoptosis 2 (Diap2)**	apoptosis	RNAi	UAS-Diap2-shRNA (Short Hairpin HMS00085)	B-34476				wildtype
**defective proboscis extension response 5 (dpr5)**	unknown	RNAi	UAS-dpr5-lhRNA (Long Hairpin JF03306)	B-29627	PP 5%, LP 5%	90%	0%	wildtype
**east**	Cell death, delays cell death of salivary glands & muscles	Protein OE	UAS-eastN2(1-1520)-GFP	MW		98%	0%	wildtype

Gene pertubed	Biological function	Gene perturbation Type	Construct/ TRip #	Stock Number	Lethality Stage	Eclosion [%]	Flightless rate [%]	DIOM phenotype
**east**	Cell death, delays cell death of salivary glands & muscles	Protein OE	UAS-eastN1(1-1902)-GFP	MW		65%	100%	abnormal morphology, delays DEOM histolysis
**east**	Cell death, delays cell death of salivary glands & muscles	RNAi	UAS-east-shRNA Short Hairpin HMS00816)	B-33879	BP+PP 100%	0%	n.d.	n.d.
**Ecdysone Receptor (EcR)**	Master regulator of metamorphosis	RNAi	UAS-EcR-RNAi	B-9326	BP+PP 100%	0%	n.d.	n.d.
**Ecdysone-induced protein 63E (Eip63E)**	Metamorphosis	RNAi	UAS-Epi63E-shRNA, Short Hairpin HMS00569)	B-34075	EP 20%	80%	0%	wildtype
**Endophilin B (EndoB)**	Membrane organization	RNAi	UAS-EndoB-shRNA (Short Hairpin HMS01285)	B-34935				wildtype
**escargot (esg)**	Transcription factor	RNAi	UAS-esg-shRNA (Short Hairpin HMS00025)	34063	LP 35%	65%	partial	wildtype
**forkhead box, sub-group O (foxo)**	insulin receptor signalling, muscle atrophy	Protein OE	UAS-foxo	B-9575	BP+PP 100%	0%	n.d.	n.d.
**forkhead box, sub-group O (foxo)**	insulin receptor signalling, muscle atrophy	RNAi	UAS-foxo-shRNA (Short Hairpin HMS00422)	B-32427				wildtype
**GXIVsPla2**	phospolipase A2	RNAi	UAS-GXIVsPLA2-shRNA (Short Hairpin HMS00918)	33961	PP 9%, EP 26%, LP44%	21%	100%	abnormal morphology
**hippo (hpo)**	Hippo pathway, organ size, proliferation	RNAi	UAS-Hippo-RNAi	33614				
**hippo (hpo)**	Hippo pathway, organ size, proliferation	RNAi	UAS-hpo-shRNA (Short Hairpin GL00046)	B-35176		100%	0%	wildtype
**happyhour (hppy)**	Cell death, size and growth	RNAi	UAS-hppy-shRNA (Short Hairpin GL00185)	B-35284		100%	0%	wildtype
**Heterogeneous nuclear ribonucleoprotein at 98DE (Hrb98DE)**	mRNA splicing	RNAi	UAS-Hrb98DE-shRNA (Short Hairpin HMS00342)	B-32351	EP 25%, LP 40%	35%	100%	abnormal morphology

Gene pertubed	Biological function	Gene perturbation Type	Construct/ TRip #	Stock Number	Lethality Stage	Eclosion [%]	Flightless rate [%]	DIOM phenotype
**Insulin-like receptor (InR)**	cell survival, body and organ size	RNAi	UAS-InR-shRNA (Short Hairpin GL00139)	B-35251	PP 5%, EP 80%, LP 10%	5%	yes	atrophy
**immune response deficient 1 (ird1)**	Autophagy	RNAi	UAS-ird1-shRNA (Short Hairpin GL00085)	B-35209				wildtype
**Lola-like (lolal)**	chromatin silencing	RNAi	UAS-lolal-shRNA (Short Hairpin GLV21087)	B-35722	LP 5%	95%	0%	wildtype
**Lst8**	Cell size, TOR signalling	RNAi	UAS-Lst8-shRNA (Short Hairpin HMS01350)	B-34361				wildtype
**muscleblind (mbl)**	muscle development	RNAi	UAS-mbl-lhRNA (Long Hairpin JF03264)	B-29585				wildtype
**misfire (mfr)**	Ferlin domain	RNAi	UAS-mfr-shRNA (Short Hairpin GLV21054)	B-35689	LP 25%	75%	No	wildtype
**Molecule interacting with CasL (Mical)**	sarcomere organization, actin depolymerization	RNAi	UAS-Mical-lhRNA (Long Hairpin JF01625)	B-31148		100%	No	wildtype
**modifier of rpr and grim, ubiquitously expressed (morgue)**	cell death	RNAi	UAS-morgue-RNAi	VDRC 11090	LP 7%	93%	0%	wildtype
**Muscle protein 20 (Mp20)**	muscle differentiation	RNAi	UAS-Mp20-shRNA (Short Hairpin HMS00630)	B-34963				wildtype
**Msp300 (Muscle specific protein 300)**	muscle differentiation	RNAi	UAS-Msp300-shRNA (Short Hairpin HMS00368)	B-32377				wildtype
**myotubularin (mtm)**	myotubularin	RNAi	UAS-mtm-shRNA (Short Hairpin HMS01806)	B-38339				wildtype
**Megator (Mtor)**	mitosis, chromatin, nuclear pore complex	RNAi	UAS-Mtor-shRNA (Short Hairpin HMS00735)	B-32941	BP+PP 100%	0%	n.d.	n.d.
**Myo31DF (Myosin 31DF)**	movement of organelles	RNAi	UAS-Myo31DF-shRNA (Short Hairpin HMS00928)	B-33971		100%	0%	wildtype
**p53**	cell death	RNAi	UAS-p53-shRNA (Short Hairpin GL01032)	B-36814		100%	0%	wildtype
**p53**	cell death	RNAi	UAS-p53-shRNA (Short Hairpin HMS02286)	B-41720	EP 5%	95%	0%	wildtype
**Polycomblike (Pcl)**	Transcription factor	RNAi	UAS-Pcl-shRNA (Short Hairpin HMS00897)	B-33946		100%	0%	wildtype

Gene pertubed	Biological function	Gene perturbation Type	Construct/ TRip #	Stock Number	Lethality Stage	Eclosion [%]	Flightless rate [%]	DIOM phenotype
**Phosphofructokinase (Ppfk)**	Metabolism	RNAi	UAS-Pfk-shRNA (Short Hairpin HMS01324)	B-34336		100%	100%	inconclusive
**Phosphofructokinase (Pfk)**	Metabolism	RNAi	UAS-Pfk-shRNA (Short Hairpin GL00298)	B-36782		100%	0%	wildtype
**Phosphotidylinositol 3 kinase 59F (Pi3K59F)**	autophagy	RNAi	UAS-Pi3K59F-shRNA (Short Hairpin GL00175)	B-36056				wildtype
**cAMP-dependent protein kinase 1 (Pka-C1)**	protein kinase	RNAi	UAS-Pka-C1-shRNA (Short Hairpin GL00038)	B-35169		100%	0%	wildtype
**pelle (pll)**	cell death	RNAi	UAS-pll-shRNA (Short Hairpin HMS01213)	B-34733		100%	0%	wildtype
**pelle (pll)**	cell death	RNAi	UAS-pll-shRNA (Short Hairpin GL00150)	B-35577		100%	0%	wildtype
**Rac1**	Muscle cell differentiation	Protein OE (CA)	UAS-Rac1.V12	B-6291	BP 100%	0%	n.d.	n.d.
**Rac1**	Muscle cell differentiation	Protein OE (DN)	UAS-Rac1.N17	B-6292	EP 50%, LP 17%	33%	100%	inconclusive
**target of rapamycin (TOR)-associated protein (raptor)**	TOR signalling	RNAi	UAS-raptor-shRNA (Short Hairpin HMS00124)	B-34814	PP 100%	0%	n.d.	n.d.
**Ras**	cell death and proliferation	Protein OE (DN)	UAS-Ras.N17	B-4846		100%	0%	wildtype
**Ras oncogene at 64B (Ras64B)**	cell death and proliferation	Protein OE (CA)	UAS-Ras64B.V14	B-2025		100%	100%	wildtype
**Ras oncogene at 85D (Ras85D)**	cell death and proliferation	Protein OE	UAS-Ras85D	B-5788		100%	0%	wildtype
**Ras85D**	cell death and proliferation	Protein OE (CA)	UAS-Ras.V12	B-4847	BP 100%	0%	n.d.	n.d.
**Ras85D**	cell death and proliferation	Protein OE (DN)	UAS-Ras85D.N17	B-4845		100%	0%	wildtype
**reaper (rpr)**	cell death	Protein OE	UAS-rpr	B-5824	BP 100%	0%	n.d.	n.d.
**Ras homolog enriched in brain ortholog (Rheb)**	TOR pathway	Protein OE	UAS-Rheb (3rd)	B-9689		100%	partial	wildtype

Gene pertubed	Biological function	Gene perturbation Type	Construct/ TRip #	Stock Number	Lethality Stage	Eclosion [%]	Flightless rate [%]	DIOM phenotype
**Ras homolog enriched in brain ortholog (Rheb)**	TOR pathway	RNAi	UAS-Rheb-shRNA (Short Hairpin HMS00923)	B-33966	LP 19%	81%	100%	atrophy
**Rho1**	actin cytoskeleton	Protein OE (DN)	UAS-Rho1.N19	B-7328	LP 70%	30%	0%	n.d.
**rapamycin-insensitive companion of Tor (rictor)**	TOR pathway	RNAi	UAS-rictor-shRNA (Short Hairpin GL00544)	B-36584	LP 5%	95%	33%	wildtype
**rapamycin-insensitive companion of Tor (rictor)**	TOR pathway	RNAi	UAS-rictor-shRNA (Short Hairpin HMS01588)	B-36699		100%	0%	wildtype
**RPS6-p70-protein kinase (S6k)**	protein translation	RNAi	UAS-S6k-shRNA (Short Hairpin GL01327)	B-41895				wildtype
**spalt-major (salm)**	Muscle cell differentiation, myofibre switch	RNAi	UAS-salm-RNAi (Short Hairpin HMS00594)	B-33714		100%	0%	wildtype
**salvador**	Hippo pathway, organ size, proliferation	RNAi	UAS-salvador-shRNA (Short Hairpin HMS00760)	B-32965			0%	wildtype
**scarface (scaf)**	proteolysis	RNAi	UAS-scaf-lhRNA (Long Hairpin JF03318)	B-29386		100%	0%	wildtype
**seizure (sei)**	potassium channel	RNAi	UAS-sei-lhRNA (Long Hairpin JF01474)	B-31681		100%	0%	wildtype
**Skeletor**	spindle matrix	RNAi	UAS-Skeletor-lhRNA (Long Hairpin JF01407)	B-31622		100%	0%	wildtype
**small ribonucleoprotein particle U1 subunit C (snRNP-U1-C)**	mRNA splicing	RNAi	UAS-snRNP-U1-C-shRNA (Short Hairpin HMS00137)	B-34822	BP 100%	0%	n.d.	n.d.
**thin, another B-box affiliate (tn)**	muscle development	RNAi	UAS-tn-shRNA (Short Hairpin HMS02508)	B-42826			0%	wildtype
**Target of rapamycin (TOR)**	TOR pathway, cell size control	Protein OE	UAS-TOR	B-7012	PP 85%, EP 10%, LP 5%	0%	n.d.	n.d.
**Target of rapamycin (TOR)**	TOR pathway, cell size control	RNAi	UAS-TOR-shRNA (Short Hairpin GL00156)	B-35578	LP 20%	80%	partial (29%)	atrophy

Gene pertubed	Biological function	Gene perturbation Type	Construct/ TRip #	Stock Number	Lethality Stage	Eclosion [%]	Flightless rate [%]	DIOM phenotype
**Target of rapamycin (TOR)**	TOR pathway, cell size control	RNAi	UAS-TOR-shRNA (Short Hairpin HMS01114)	B-34369		100%	0%	wildtype
**Target of rapamycin (TOR)**	TOR pathway, cell size control	RNAi	UAS-TOR-shRNA (Short Hairpin HMS00904)	B-33951	PP 100%	0%	n.d.	n.d.
**terribly reduced optic lobes (trol)**		RNAi	UAS-trol-shRNA (Short Hairpin GL01153)	B-42783				wildtype
**Tsc1**	TOR pathway, cell size control	RNAi	UAS-Tsc1-shRNA (Short Hairpin GL00012)	B-35144		80%	22%	Hypertrophy
**Tsc2, gigas (gig)**	TOR pathway, cell size control	RNAi	UAS-Tsc2-shRNA (Short Hairpin HMS01217)	B-34737	PP 20%, EP 5%, LP 15%	60%	64%	hypertrophy
**Tsc2, gigas (gig)**	TOR pathway, cell size control	RNAi	UAS-Tsc2-shRNA (Short Hairpin GL00321)	B-35401		100%	6%	Hypertrophy
**Ubiquitin conjugating enzyme (UbcD6)**	Proteolysis	RNAi	UAS-UbcD6-shRNA (Short Hairpin GL00405)	B-35476	LP 5%	95%	0%	wildtype
**Unc-89**	sarcomere organization, muscle differentiation	RNAi	UAS-Unc-89-shRNA (Short Hairpin HMS00963)	B-34000		100%	0%	wildtype
**UV-resistance associated gene (UVRAG)**	Autophagy, Cell growth	RNAi	UAS-Uvrag-shRNA (Short Hairpin HMS01357)	B-34368				Hypertrophy
**warts (wts)**	Hippo pathway, organ size, proliferation	RNAi	UAS-wts-shRNA (Short Hairpin HMS00026)	B-34064		100%	0%	wildtype
**yorkie (yki)**	Hippo pathway, organ size, proliferation	RNAi	UAS-yki-shRNA (Short Hairpin HMS00041)	B-34067	LP 7%	93%	57%	atrophy
